# Splenic lymphangioma in adulthood: A case report

**DOI:** 10.1016/j.ijscr.2020.01.061

**Published:** 2020-02-11

**Authors:** Anthony Perez, Mary Ellen C. Perez, Ann Camille Yuga, Brent Andrew G. Viray

**Affiliations:** a Department of Surgery, University of the Philippines-Philippine General Hospital, Philippines; b Division of Hepatopancreaticobiliary Surgery, Department of Surgery, University of the Philippines-Philippine General Hospital, Philippines; c Department of Anesthesiology, University of the Philippines-Philippine General Hospital, Philippines

**Keywords:** Splenic lymphangioma, Adulthood, Laparoscopic splenectomy

## Abstract

**Introduction:**

Splenic disease are uncommon and primary tumors of the spleen are very rare. These are classified as splenic cyst, benign and malignant tumors. Splenic lymphangioma is a rare, slow-growing benign tumor of the spleen. More often seen in children, it is a congenital malformation of the lymphatic system and is rarely seen in adulthood. When present in adults, it is usually asymptomatic and would be incidentally detected through imaging studies. Infrequently, some patients would present with abdominal pain, distension, nausea, and may have palpable abdominal mass. It may be solitary or may have multiple splenic lesions.

**Presentation of case:**

We present of a case of splenic mass in a 56-year old female, which remained undetected until incidentally discovered on work-up for chronic back pain. Laparoscopic splenectomy was eventually performed with eventual resolution of the chronic back pain. Histopathologic examination revealed splenic lymphangioma.

**Discussion:**

Splenic tumors are uncommon. Splenic lymphangiomas presenting in adulthood are extremely rare, and preoperative diagnosis may be difficult in asymptomatic patients.

**Conclusion:**

Surgical removal of splenic lymphangiomas may be necessary to relieve symptoms, prevent complications and confirm diagnosis.

## Highlights

• Splenic diseases are uncommon and primary tumors of the spleen are very rare.
• Splenic lymphangioma is a rare, slow-growing benign tumor of the spleen which are rare in children but more so in adulthood.
• When present in adults, it is usually asymptomatic and would be incidentally detected through imaging studies.
• Splenic lymphangiomas may require splenectomy when symptomatic, to resolve diagnostic dilemmas or prevent complications.

## 1 Introduction

Splenic diseases are uncommon and primary tumors of the spleen are very rare. Splenic tumors are classified as splenic cysts, benign and malignant tumors. Splenic lymphangioma is a rare, disease of the lymphatic system and accounts for <0.007% of all tumors [1,2]. It a slow-growing neoplasm usually seen during childhood and rarely seen beyond 20 years of ae [3]. If present in adulthood, is usually an incidental finding. However, due to increase in the size of the spleen, some adult patients may present with left upper quadrant pain, nausea, abdominal distention and palpable abdominal mass [4,5]. This case is being present due to the rarity of this condition. This is the first reported case in our tertiary government institution and our developing country in general. We are reporting the diagnostic approach and the management, reporting in line with the statement: Updating Concensus Surgical Case Report Guidelines (SCARE) [6].

## 2 Case

**Fig. 1:**
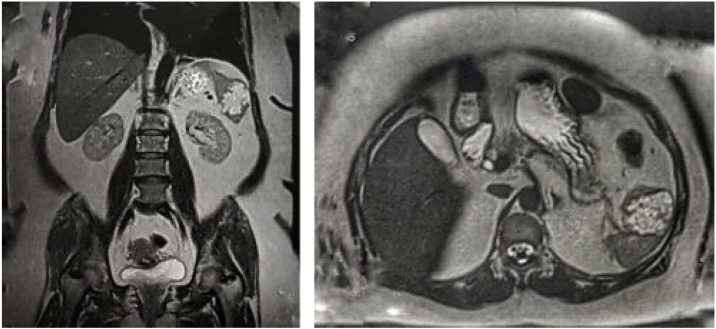
Noted splenic mass, approximately 5 cm in the patient’s MRI which appeared hyperintense in T1-weighted images, implying the fluid contents of the mass.

A 56-year old woman presented with chronic back pain. No history of trauma and no other associated symptoms. Patient’s personal and family medical histories did not show positive signs and symptoms of any splenic pathology. Physical examination likewise did not show any palpable splenic mass or splenomegaly. Laboratory workup was within the normal limits. She underwent thoracolumbar MRI to investigate her chronic back pain which incidentally showed 5 cm splenic mass which appeared hyperintense on T1-weighted images (Fig. 1).

**Fig. 2:**
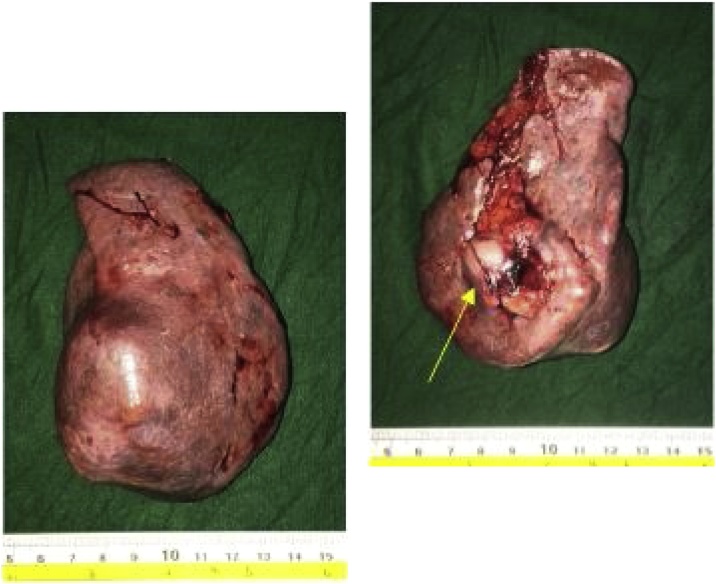
A and B. Patient’s spleen with notable mass (yellow arrow) at its medial aspect 3.8 × 3.2 × 4.2 cm mass predominantly cysts containing serous and mucinous fluid.

She was admitted in our institution and was subsequently prepared for laparoscopic splenectomy. Preoperative cardiopulmonary screening did not reveal any significant comorbidities. She had previous immunization as per established protocols for elective splenectomy. On surgery under general anesthesia, she was positioned in right lateral semi-decubitus position. A 10 mm Hasson trocar was inserted through the left periumbilical area followed by CO2 insufflation. Three more trocars (10 mm, two 5 mm trocars) were inserted at the left subcostal area. Abdominal inspection was done. Splenic artery and vein were identified and doubly ligated and clipped. Afterwards, splenocolic, lienorenal and phrenicosplenic ligaments were dissected and proceeded with ligation of the short gastric vessels. The spleen was then extracted. Grossly, the spleen was enlarged to 6 × 4 × 10 cm (Fig. 2A and 2B) and weighed 380 g with well-defined 3.8 × 3.2 × 4.2 cm mass predominantly cysts measuring less than 5 mm in diameters and containing serous and mucinous fluid. No solid or complex areas were identified. The rest of the splenic parenchyma is normal. Perioperative course was uneventful and the chronic back pain resolved. Final histopathological result was splenic lymphangioma.

## 3 Discussion

Splenic lymphangioma is a benign cystic neoplasm that resulted from congenital malformation of the lymphatic system and accounts for <0.007% of all tumors [1,2]. It is a slow-growing neoplasm usually seen during childhood and rarely seen beyond 20 years of age [3]. If present in adulthood, it is usually an incidental finding. In our patient, the chronic back pain could have been attributed to the splenic lesion. It is a rare case, presenting in a 56-year old and being virtually undiagnosed if not for consulting for a seemingly unrelated symptom. Symptoms in patients with splenic lymphangioma are usually due to increase in size of the spleen. Patients may present with left upper quadrant paint, nausea, abdominal distension and palpable abdominal mass [4,5]. Lymphangiomas in general, most commonly involve the neck (75%), axilla (20%) and less common in the mediastinum, retroperitoneum, kidney, bone, adrenals, spleen, liver and pancreas. When it involves several sites it presents as lymphangiomatosis syndrome [3,9]. There are two hypotheses for the formation of lymphangioma of the spleen: (1) congenital malformation of the spleen (2) inflammation of the lymphatic system causing obstruction and consequently formation of lymphangioma [5].

Since it is frequently undetected clinically, its diagnosis is often made through imaging such as abdominal ultrasonography, abdominal computed tomography (CT) scan or magnetic resonance imaging (MRI) [5,7]. Our patient was advised to undergo the MRI mainly to evaluate her lumbosacral spine, thereby detecting the cystic lesion. These cystic lesions of varying sizes appear as well-defined hypoechoic masses with internal septations and occasional echogenic debris within the fluid-filled loculi on ultrasonography. On CT scan, these well-demarcated cysts typically found in the subcapsular regions appear hypodense with few enhancing septa and occasional peripheral rim calcification. On MRI, it usually appears as well-defined multilocular cystic lesions with thin septations homogenously hypointense on T1-weighted images. On T2-weighted images, it appears hyperintense when filled with hemorrhagic or proteinaceous material. However, on certain occasions, it may also show high signal intensity on T1-weighted images attributed to the proteinaceous or hemorrhagic contents similar to the case presented (Fig. 1) [[8], [9], [10]]. These cystic lesions are avascular on angiography [11].

**Fig. 3:**
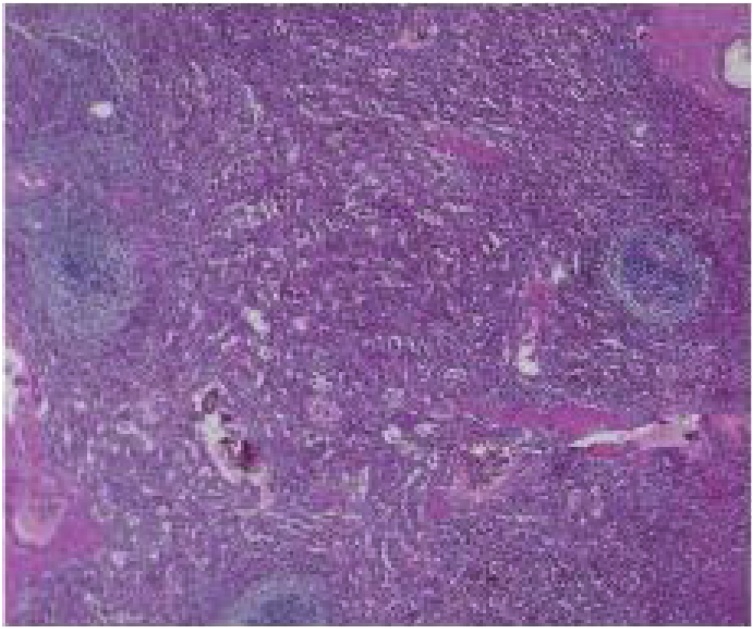
Normal spleen.

A normal spleen would appear as a dark red to blue-black organ at the left upper quadrant of the abdomen (Fig. 3). It is covered with capsule composed of elastic fibers, dense fibers and smooth muscles encasing the splenic parenchyma, which in turn is made up of red pulp and white pulp. The red pulp is a network of splenic cords and venous sinuses which contains the erythrocytes, granulocytes, lymphocytes and mononuclear cells whereas the white pulp is made up of periarteriolar lymphoid sheath (PALS), the follicles and the marginal zone [12]. In contrast, grossly, a splenic lymphangioma, may appear as a solitary or multicystic nodules or sometimes diffuse lymphangiomatosis with minimal normal splenic parenchyma. It may have single thick-walled cyst or multiple thin-walled cyst of varying sizes containing pinkish or clear fluid [10].

**Fig. 4:**
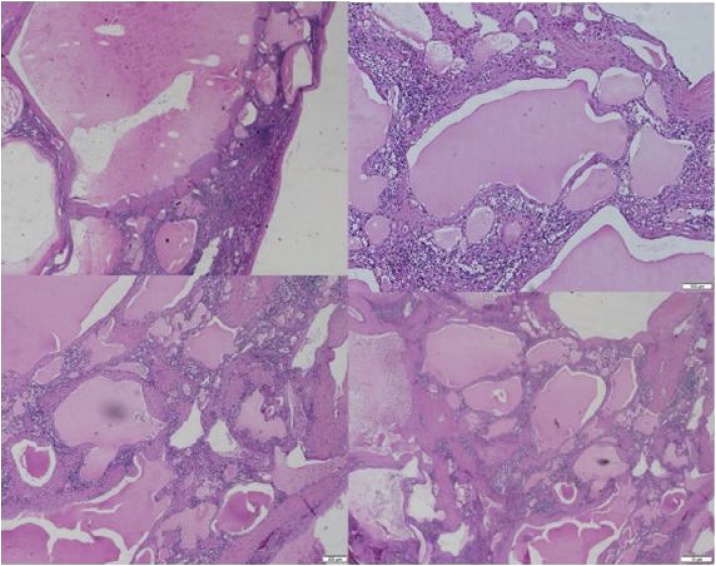
Histopathology of the patient’s splenic mass showing cystic lymphangioma of the spleen and its usual location in the subcapsular region. Note the lymphatic fluid within the septations, which appear as eosinophilic amorphous proteinaceous material.

Microscopically, these cysts are made up of multiple vascular channels lined by single layer of endothelial cells and contain eosinophilic amorphous proteinaceous contents (Fig. 4) [7]. Lymphangiomas are classified into 3 types according to the size of the dilated lymphatic channels: (1) capillary (supermicrocystic) (2) cavernous (microcystic) (3) cystic (macrocystic) [5,7,10,11]. Cystic lymphangiomas just like in this case are the most common variant and it appears as a honeycomb of varying sizes of thin-walled cysts containing lymph [10,11]. The remaining parenchyma may or may not show fibrosis, inflammation or congestion. Selective markers such as podoplanin (D2-40), a monoclonal antibody against dysgerminoma, can be used to distinguish lymphangiomas from hemagiomas as it selectively stains the lymphatic endothelium [10,11]. Other immunohistochemical techniques such as CD31, CD34 and factor VIII can likewise be utilized [10].

Surgical management including both total and partial splenectomy is recommended while conservative treatment is still on debate [1,5]. Laparoscopic splenectomy such as in our case is increasing in popularity especially in normal to moderately enlarged spleen. Other therapeutic strategies such as irradiation, drainage and aspiration may show little success [11]. Laparoscopic splenectomy has been widely accepted technique for removal of spleen. Similar to other laparoscopic procedures, laparoscopic splenectomy has found to have better outcomes and can minimize postoperative complications. Contraindication to its use is a massive splenomegaly [10]. In performing this procedure, patient can be positioned either in anterior, semi-decubitus or lateral position. Most commonly used position is the semi-decubitus, like in the presented case, wherein the patient is in the right lateral decubitus position at 45 degrees. This manner is the preferred position by the surgeons because it makes it easier for them to gain access to the posterior aspect of the spleen,its ligaments as well as to the short gastric vessels. Hilar vessels can be easily ligated without the danger of causing injury to the pancreas [1,14].

The use of laparoscopic technique warrants careful preoperative planning with consideration of the size and volume of the spleen. Like in other operations, laparoscopic splenectomy entails complications, most common of which is bleeding. Among other complications of this procedure are pancreatitis, ileus, abdominal wall infections/hematoma, pneumonia and atelectasis and overwhelming postsplenectomy infection (OPSI). Included in postoperative planning is the administration of vaccines (*HiB*, *meningococcal* and *pneumococcal*). But with further improvement with the laparoscopic tools as well as more experience by the surgeons, this technique may be considered the future standard procedure for all types of splenectomy [14]. Malignant transformation is very low and likewise the recurrence rate, which is approximately 9.5%, usually after incomplete resection. Other complications of this condition such as infection, torsion, bleeding and rupture may be avoided with immediate surgical management once diagnosis has been established [1,13]. Its prognosis after complete resection is favorable [1,5,11].

## 4 Conclusion

Splenic tumors are uncommon, splenic lymphangiomas presenting in adulthood are extremely rare, and preoperative diagnosis may be difficult in asymptomatic patients. Once diagnosed, surgery may be the most effective modality of treatment and laparoscopic splenectomy may avoid potential complications.

## Declaration of Competing Interest

No financial or personal disclosures.

## Funding

No external source of funding needed for the case report.

## Ethical approval

This case report was approve d and registered with the ethics board of the University of the Philippines. No issues in the approval of the report were encountered.

## Consent

No patient identifiers were included in the study and full informed consent was obtained.

## Author contribution

Anthony R. Perez MD, MHA: Conceptualization; Data curation; Formal analysis; Funding acquisition; Investigation; Methodology; Project administration; Roles/Writing - original draft; Writing - review & editing.

Mary Ellen Chiong Perez MD: Resources; Software; Supervision; Validation; Visualization; Roles/Writing - original draft; Writing - review & editing.

Ann Camille Yuga MD: Data curation; Formal analysis, Investigation; Methodology; Resources; Software;Validation; Visualization; Roles/Writing - original draft.

Brent Viray MD: Data curation; Formal analysis;Investigation; Resources; Writing - review & editing.

## Registration of research studies

Registration No. RGAO-2018-0542 Research Grants Administration Office, University of the Philippines Manila.

## Guarantor

Anthony R. Perez, MD, MHA.

## Provenance and peer review

Not commissioned, externally peer-reviewed.
